# Three years follow-up after cryoablation of a right atrial myxoma arising from the Koch’s triangle

**DOI:** 10.1186/s13019-016-0548-2

**Published:** 2016-11-22

**Authors:** P. Wauthy, D. Mircev, S. Marinakis

**Affiliations:** 1 Department of Cardiac Surgery, CHU Brugmann, Université Libre de Bruxelles, Place Van Gehuchten 4, 1020 Brussels, Belgium; 2 Department of Cardiology, Iris Sud Hospitals, Rue Jean Paquot 63, 1050 Brussels, Belgium; 3 Department of Cardiac Surgery, Hospital Civil Marie Curie, Chaussée de Bruxelles 140, 6042 Charleroi, Belgium

**Keywords:** Myxoma, Cardiac tumors, Koch’s triangle, Cryoablation

## Abstract

We reported 3 years ago the use of cryoablation in the treatment of a right atrium myxoma arising from the Koch’s triangle. The atrioventricular conduction was successfully preserved. Today, after 3 years follow-up, the patient remains with a conducted sinus rhythm and is free of recurrence. Even if extensive resection of the stack of the myxoma remains the first choice attitude, cryoablation could be considered as a serious second choice alternative.

## Dear editor

**Fig. 1:**
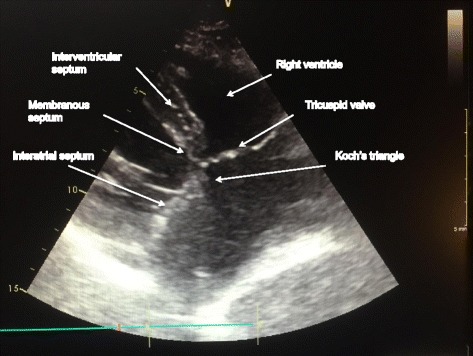
Transthoracic echocardiography demonstrating the absence of recurrence in the right atrium close to the Koch’s triangle

We reported 3 years ago the treatment of a right atrial myxoma arising from the Koch’s triangle [1]. To preserve the atrioventricular conduction, we limited the excision of the myxoma without extensive stalk resection and we additionally applied cryoablation to reduce the risk of secondary recurrence. Despite immediate success on atrioventricular conduction, questions remains on long term evolution of the conduction and potential recurrence of the tumor. Three years after surgical resection of the tumor, based on an ultimate echocardiography (Fig. 1) and electrocardiogram at the beginning of 2016, we can state that the patient remains with preserved atrioventricular conduction and is free of tumor recurrence.

In the literature, only one case of cryoablation in complement to surgical resection is reported [2]. This case involved a left ventricular myxoma recurrence 2 years after initial surgical resection and the use of cryoablation in complement to surgical resection. Unfortunately, the follow-up of this patient is limited to 1 year. Wang et al. [3] reported recently in an important series an overall rate of recurrence of 5%. Recurrences of cardiac myxomas after resection have been for a long time mainly attributed to incomplete resection of the myxoma’s pedicle [4, 5]. Nowadays, even if this consideration remains relevant, multicentric myxomas growth is considered as the principal risk factor for recurrence [3]. Shinfeld et al. analyzed the reported cases of recurrent myxomas and observed that up to 57% of them were diagnosed within the first 3 years following initial treatment [6]. Finally, Gerbode et al. reported that myxomas recurrence occurs preferably in men than in women [7]. After all these considerations, we can reasonably state that this 81 years old women will not likely present a local recurrence of the initial myxoma. Though we conclude that even if extensive stack resection remains the golden standard attitude, cryoablation may be considered as a valuable secondary alternative to extensive myxomas stalk resection when this excision is hazardous.
